# Community-Engaged Data Science (CEDS): A Case Study of Working with Communities to Use Data to Inform Change

**DOI:** 10.1007/s10900-024-01377-y

**Published:** 2024-07-03

**Authors:** Ramona G. Olvera, Courtney Plagens, Sylvia Ellison, Kesla Klingler, Amy K. Kuntz, Rachel P. Chase

**Affiliations:** 1 The Center for the Advancement of Team Science, Analytics, and Systems Thinking (CATALYST), College of Medicine, The Ohio State University, 700 Ackerman Road, Suite 4000, Columbus, OH 43202 USA; 2 College of Medicine, HEALing Communities Study, The Ohio State University, Columbus, OH USA; 3 Research Information Technology, College of Medicine, The Ohio State University, Columbus, OH USA

**Keywords:** Data driven decision making, Community-based participatory research, Community-engagement, Data sharing

## Abstract

Data-informed decision making is a critical goal for many community-based public health research initiatives. However, community partners often encounter challenges when interacting with data. The Community-Engaged Data Science (CEDS) model offers a goal-oriented, iterative guide for communities to collaborate with research data scientists through data ambassadors. This study presents a case study of CEDS applied to research on the opioid epidemic in 18 counties in Ohio as part of the HEALing Communities Study (HCS). Data ambassadors provided a pivotal role in empowering community coalitions to translate data into action using key steps of CEDS which included: data landscapes identifying available data in the community; data action plans from logic models based on community data needs and gaps of data; data collection/sharing agreements; and data systems including portals and dashboards. Throughout the CEDS process, data ambassadors emphasized sustainable data workflows, supporting continued data engagement beyond the HCS. The implementation of CEDS in Ohio underscored the importance of relationship building, timing of implementation, understanding communities’ data preferences, and flexibility when working with communities. Researchers should consider implementing CEDS and integrating a data ambassador in community-based research to enhance community data engagement and drive data-informed interventions to improve public health outcomes.

**Supplementary Information:**

The online version contains supplementary material available at 10.1007/s10900-024-01377-y.

## Introduction

Data are critical for making informed decisions, yet many community-based organizations and local government agencies lack funding and resources, such as technology and access to data scientists who can perform data management and analysis for strategic planning and decision making. This dearth of resources to support the use of data to drive decision making is ubiquitous in community groups and multiple sector coalitions that are crafting their own decision-making tools [1]. This is particularly apparent during emergencies and crises, when accurate data and data analysis can facilitate communities and organizations in prioritizing resources and target interventions to strive for optimal outcomes. For example, the data needed for planning and outreach during health-related crises, such as the HIV/AIDS epidemic [2] and the COVID-19 pandemic [3, 4] aided cross-sector coordinated community efforts to reach vulnerable populations.

The ongoing opioid epidemic is a current health emergency [5] that warrants cross-sector community-based planning using data. With more than 100,000 overdose deaths per year in the United States, mainly from opioids [6], communities are seeking support to decide among and implement various evidence-based practices (EBPs) that are known to be effective in alleviating overdoses and overdose deaths. Those working in the communities understand that having, sharing, and using data is valuable to make informed decisions, yet they may lack the staffing, tools, knowledge, or skills to fully engage in the planning process that is fundamental for cross-sector, community-based, data-driven decision making (DDDM) to address the opioid epidemic.

A few frameworks are available for those interested in cross-sector data planning and data sharing [6–12], yet they are not designed for community utilization to address specific needs. For example, the Data Across Sectors for Health (DASH) program promotes collaboration via multi-sector data-sharing [7, 11], but it is not itself a framework for community-driven planning. Other approaches, such as Connected Communities of Care (CCC) [12], Accountable Communities for Health (ACH) [10, 13], Community Learning through Data Driven Discovery (CL3D) [14, 15], and BUILD Health Challenge [16] exist. However, none of these models present a step-by-step framework to guide communities, particularly those with limited data expertise or data collection experience. Further, these models do not guide data scientists, who traditionally may have limited experience working with community-based organizations, to collaboratively identify and achieve community goals.

This article presents a novel model, Community-Engaged Data Science (CEDS), to bridge this gap by establishing a step-by-step guide for communities and data science researchers to capitalize on each other’s expertise. As part the NIH-funded HEALing (Helping to End Addiction Long-term^®^) Communities Study (HCS) that focused on reducing opioid overdose deaths, researchers developed CEDS to aid in centering data to support a goal-oriented, iterative process for communities to work collaboratively with data scientists to plan and implement data use and sharing to prioritize and address the opioid epidemic. We present the CEDS step-by-step guide with lessons learned from implementing CEDS as part of the HCS to support community decision-makers interested in integrating relevant data into community health improvement activities.

## Foundations of CEDS

There is a long tradition in public health of adopting best practices from other fields and disciplines for creating change [17]. The CEDS framework continues this tradition by drawing on the principles of data science and community-based participatory research (CBPR), with influence from human-centered design (HCD), to be a tool that communities can use to bring data to the forefront of goal setting, planning, and decision-making to implement measurable change grounded in and valuing the experience of cross-sector partnerships.

CBPR is an approach founded on full and equal partnership between community members, organizational representatives, researchers, and others in all aspects of the research process [18–23]. Co-researcher partners each contribute expertise and share in decision-making to study and achieve policy or social change which directly benefits community members [24, 25]. HCD uses nonlinear, iterative processes, with continual and rapid development and reassessment [26–28]. Through phases of inspiration, ideation, and implementation, HCD systematically approaches problem-solving with emphasizing community engagement to understand barriers and unmet needs, uses rapid prototyping of solutions responsive to those needs, and engages in continual community feedback to adjust the implementation process [26, 29, 30]. Data science, from computer science and technological research, focuses on the how and why data are collected, collated, organized, analyzed, and communicated [31, 32]. Data science values DDDM and accounts for the tremendous effort that is required to ensure data are accurate, meaningful, and sustainable to aid community decision-making processes and achieve community goals [31]. At its core, data science focuses on extracting meaningful evidence and knowledge from data [32].

Using concepts from CBPR, HCD, and data science, CEDS is designed as a step-by-step guide to assist communities and data scientists in collaborating to develop community data action plans (DAP). A DAP is a community-driven, bottom-up approach to DDDM and should begin with a focus on community needs and then explore how data can help address those needs. Community members under pressure to rapidly solve a problem and traditionally trained data scientists often approach this process backwards. They may feel compelled to use a top-down paradigm, starting with the identification of available high-level data sources, then figuring out what questions those data can answer. This approach is problematic for planning and decision-making because it only allows communities to: (1) apply coarser geographic level data (i.e. state or county-level) to more localized (i.e., neighborhood-level) issues and (2) use existing data collected for purposes that do not align with the community’s needs. Thinking about the problem and beginning the planning process this way hampers the community’s ability to set goals, limiting achievable goals to a small subset of issues addressed by existing data, and compromising the community’s ability to effectively address questions specific to their needs and interests.

In its partnership approach, CEDS seeks to involve all identified key community members as partners in and owners of the process, who contribute their expertise and participate in decision-making from conception through execution to dissemination of the community DAP. CEDS acknowledges there are often existing data resources, data sharing agreements, and content area and analytic expertise among community organizations which can be adapted and built upon throughout the process. The CEDS framework also provides a forum where cultural and organizational differences among participants can be openly discussed and addressed to build trust and sustainable data relationships.

CEDS embraces HCD techniques for user-centered development of metrics and data systems that can be used to continually monitor and improve processes in the community. Once communities have identified unmet data needs, CEDS promotes the collaborative design of tools such as data dashboards to meaningfully visualize community data, or data commons to share data across organizations and sectors. Data scientists can develop prototypes, share them with community for review and feedback, and then revise them as often as needed to better address community needs and utility. A key to CEDS success is this HCD-inspired iterative approach and continued community feedback that limits the extent to which a design team might put effort into product features that are a poor fit for a community, focuses efforts on features that are important to achieving community goals, and promotes continual development and maintenance of shared understandings across groups working toward common goals [29, 30]. Where the product is a DAP, this process enhances the utility of data collected or shared to achieve community goals and increases the value of investing in a tailored data system.

## Data Ambassadors and the CEDS Steps

The step-by-step CEDS model was designed to be a practical tool to assist communities and data scientists with collaboration to address community needs. However, traditionally trained data scientists and communities with less data experience and fewer resources may find it beneficial to engage and empower a data ambassador. A data ambassador acts as an interpreter or an intermediary between the community partners and data workers bridging gaps between these two groups and facilitating communication to build shared insights, goals, and processes. The data ambassador does not need the experience of a data scientist but should be comfortable with data and know (or be willing to learn) the basics of data interpretation. Additionally, data ambassadors must be skilled at community engagement and communication. The skills needed to be a data ambassador may be found within individuals already working in organizations in the community (such as a data champion) or, if part of a larger project, may be found in a dedicated team member whose job focuses on data and community-engagement.

**Fig. 1:**
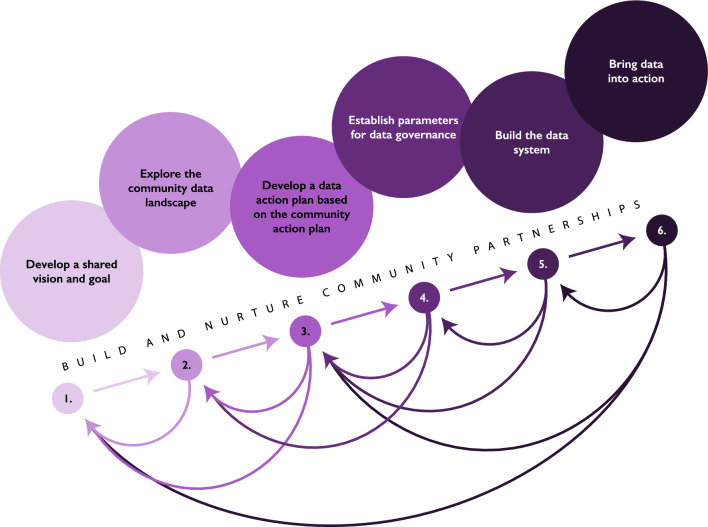
The community-engaged data science (CEDS) model

The data ambassador’s role is to guide community partners through the iterative steps of CEDS. Although presented linearly, the model is more fluid, often necessitating return to previous steps along the way. The CEDS model begins with a period of community building and partnership (Step 0) and works through six additional steps: (1) Develop a shared vision and goal; (2) Explore the community data landscape; (3) Develop a DAP based on the community action plan; (4) Establish parameters for data governance; (5) Build data systems; and (6) Bring the data into action. This step-by-step process can be used first to select appropriate strategies to tackle local problems, then again to monitor whether and how the employed strategy makes sustainable change. A visualization of the iterative steps in CEDS is presented in Fig. 1.

### Step 0: Build and Nurture Community Partnerships

Before beginning to address data, the CEDS model is predicated on community-based contributors partnering to inform the process. Ideally this can involve existing or de novo community coalitions comprising multiple organizations or individuals representing diverse perspectives, experiences, and resources who are engaged in the health concern and willing to assist in the joint process for community change. Participants in coalitions or partnerships may fluctuate over time as needs change. Data ambassadors can help in building trust with and among community-based partners as trust is essential in data sharing.

### Step 1: Develop a Shared Vision and Goal

While it is tempting for data-engaged communities and data scientists to first identify available data and then figure out what kinds of problems those data can help solve, the CEDS model first focuses on delineating community needs and goals. Decision-makers should begin with identifying the priority issues and goals that the community wishes to address, and perhaps discuss processes by which they wish to achieve those goals. Discussions may help develop a “theory of change” or “logic model” [33], including prioritized ideas to produce a “community action plan”. The community action plan outlines the steps the community partners will need to take to reach their goal(s), the resources they will need to take those steps, and the outcomes the process will produce if successful. Table 1 shows an example of a community action plan to develop an overdose fatality review board.

**Table 1:** Example of community action plan (CAP) to develop a county-level overdose fatality review board

Target population	• People who use opioids or other drugs • Community services and agencies who engage with people who use drugs
Resources	• County coalition • HEALing Communities Study (HCS) • County Health Department (HD) • Alcohol, Drug Addiction Mental Health Services (ADAMHS) • Recovery Agencies • Emergency medical service (EMS) • Social Service Agencies
Activities	• Quarterly meeting of key stakeholders to discuss drug overdose deaths • Review of all relevant information on decedent’s demographics and drug use/treatment history • Develop recommendations based on analysis of gaps in care and opportunities for community improvement
Short-term outcomes	• Establish an active overdose fatality review board that meets regularly
Long-term outcomes	• Improve treatment, healthcare, social service, and other community-based systems to reduce opioid and other drug deaths
End goals	• Overdose fatality review helps reduce opioid overdose deaths

Even though this is a community-focused activity, data ambassadors should be a part of this conversation, at least as listeners, to fully understand the community’s perspectives, concerns, and goals. This early engagement is important to build trust, establish good working partnerships, and develop mutual understanding [19, 24] and may lead to “inspiration” [29, 30] for next HCD-informed steps. We emphasize that this step must be underway before the data-driven aspects of the CEDS model are employed. The community action plan is a living document, as the CEDS model is iterative and cyclical; the vision and goals of the community need not be finalized to start the next steps but will inform what comes next.

### Step 2: Explore the Community Data Landscape

Some communities may already engage in very sophisticated data-related activities, while others may not use data at all. CEDS emphasizes the importance of understanding the specific community data context, including current data resources, current activities that use data, and general community perspectives and interest in data-related activities. Data ambassadors can support the coalition in surveying the data landscape, through dialogue with key data players, to understand what data are immediately available, what data might be imminently available, and what gaps in data resources and activities could be addressed through new data endeavors.

Assessing the community data landscape will generally include careful evaluation and classification of all data sources according to the availability, frequency of data collection, reliability, representativeness, ownership, information provided, and other qualities to support the use of those data. Incorporating a formal data inventory tool (see Supplementary material 1 for example) is helpful in organizing and communicating the data landscape back to community organizations and stakeholders. Accurately depicting the landscape of existing data sharing and coordination efforts is vital to establishing community buy-in because it builds on extant foundations [34].

After mapping the community’s data landscape, the data inventory can be compared to the data needs highlighted in the community action plan, thereby identifying data opportunities and gaps. Some communities with large and complex systems may want to engage the data ambassador in further tasks related to connecting, maintaining, nurturing, and refining data networks in the community as a part of sustaining or improving a robust, comprehensive data landscape. Other communities might wish to establish a data workgroup to focus on issues surrounding data. While this can be an efficient and effective approach, data engagement is strongest when all partners experience data as part of the overall workflow rather than leaving all data activities with highly experienced data experts or in specialized silos.

### Step 3: Develop a DAP Based on the Community Action Plan

The community action plan drives the development of a DAP based on community goals. The data ambassador can help the coalition develop a DAP that defines metrics, identifies data, sets benchmarks, and establishes a strategy on how data will be shared. Ideally, the DAP has two components. First, the DAP delineates what data will be collected, how it will be managed, reviewed, and used, for what purpose, and for how long. The DAP may also reflect community decisions about who maintains the data, who will sustain the data tools or systems that are developed, how long the data collection effort will continue, and/or efforts to seek funding through grants or other community funds. The second component of a DAP maps out a course of community action in response to what the data show, including what happens when goals are reached or fail to be reached. As part of the iterative process of CEDS, crafting the DAP may drive revisions to goals or objectives, even at the planning stage, prompting stakeholders to revisit the community action plan (CEDS Step 1). See Table 2 for an example DAP that expands on the community action plan to develop an overdose fatality review board in Table 1.

**Table 2:** Example of Data Action Plan (DAP) for supporting a new county-level overdose fatality review board

Metric	Temporality	Source	Original Data System	Collectors/Data Entry into REDCap	Analyzers	When reviewed	Uses	Disseminators
Total fatal overdoses—Annual	Yearly	State Department of Health	State website	Coroner, OFR Board, DH	OFR Board, DH, HD	Quarterly through the OFR	Tracking outcomes	Coalition, DH, HD
Total # of decedents	Quarterly	Coroner’s records	Excel	Coroner, OFR Board, DH	OFR Board, DH, HD	Quarterly through the OFR	Tracking outcomes	Coalition, DH, HD
Total # of decedents by key demographics (neighborhood, income, employment status, education)	Recorded during death investigation, reported quarterly	Coroner’s records	Excel	OFR Board	OFR Board	Quarterly through the OFR	Hot spot targets certain demographics	Coalition, HD
Total # of decedents with a criminal history	Quarterly	Public Record, common pleas court	Word Documents	OFR Board	OFR Board	Quarterly through the OFR	Hot spot targets certain demographics	Coalition, HD
Total # of decedents who completed one or more treatment programs (if known)	Quarterly	Recovery agencies/ Medicaid billing	Excel, Word, Other systems	OFR Board	OFR Board	Quarterly through the OFR	Retain rate/measure success of certain programs	Coalition, HD
Total # of decedents who were administered Naloxone	Quarterly	EMS/ Death Report	Excel	OFR Board	OFR Board	Quarterly through the OFR	Compare to those who did not receive naloxone	Coalition, HD
Toxicology Result – by drug	Quarterly	Toxicology Lab	REDCap	OFR Board	OFR Board	Quarterly through the OFR	Understand what drug caused death most frequently	Coalition, HD

*HD* local health department, *DH* state department of health, *EMS* emergency medical services, *OFR* overdose fatality review

### Step 4: Establish Parameters for Data Governance

Data ambassadors may need to facilitate community data sharing as part of the community action planning. Because of the sensitive and often misunderstood nature of much data used for community-driven change, privacy concerns need to be explicitly considered, including development of memorandums of understanding and data use agreements. Data ambassadors may need to work closely with community partners to detail and weigh the benefits, such as the reduction in duplicate efforts and cost for programs, versus barriers of the data sharing, often including upfront costs and long-term gains, start-up and maintenance resources required and created, and legal considerations. Numerous practical, technological, and information barriers, including frequent overly conservative or overly liberal misinterpretation of privacy laws and guidelines, particularly regarding healthcare data [35], need to be addressed. A well-structured DAP can promote understanding of these issues and facilitate the development of data sharing agreements, if needed.

Data sharing agreements should answer key who-what-when-where-how questions regarding data sharing and management. The monetary value of data should be represented in any data sharing agreement and the mutual economic benefits of sharing data noted and valued, if possible, to promote sustainability and expansion. In cases where data privacy concerns become a barrier, data sharing should ideally be arranged through professionals in the field who have the appropriate legal knowledge and/or ability to de-identify shared data, if required. Part of the data ambassador’s role may be to seek out partnership with organizations that have and are willing to share this legal expertise.

### Step 5: Build the Data System

Once the DAP is created, and data governance is in place, the coalition can build the data system that will facilitate data collection, management, retention, and use. Referencing the DAP, data ambassadors can guide the coalition through identification of workflow and technological tools that will support the community needs to implement and sustain the proposed data plan. This might include hiring or reorganizing a workforce; establishing a digital or paper-based data collection system; purchasing and setting up software, cloud-based file-sharing, databases, and data dashboards; establishing data flow processes; and incorporating data activities into the workflow of data users. Data ambassadors may engage data scientists in this process encouraging HCD principles, such as trial, error, and reboot [29] to rapidly develop data systems that are focused on community needs. Data scientists and data ambassadors might also work with communities to develop data system trainings that ensure data are used in a manner consistent with the DAP.

### Step 6: Bring Data into Action

While gathering and displaying data is necessary to implementing the DAP, it is not sufficient. Data’s mere existence is not enough to bring data into action; data must be organized, analyzed, and communicated through metrics and visualizations meaningful to all in the community. DAP and its practical application will likely reveal challenges and opportunities to improve, innovate solutions during implementation, and require review of the DAP (and potentially the community action plan) to fully integrate those solutions in the regular aspect of continued community decision making and ensure sustainability of the process.

The emphasis here is on workflow and maintenance. Data ambassadors may assist coalitions in assigning tasks to collect, manage, analyze, use, and disseminate data to specific people and/or groups along with a schedule of activity. Coalitions must create structures to assess whether current work assignments and schedules are appropriate and schedule regular assessments of whether community action plans, DAPs, and data activity assignments are aligned with community priorities.

### Step Summary

The seven steps of CEDS are designed to lead community and community coalitions through data action planning and implementation to enable DDDM to address community priorities and needs. CEDS as a model has been presented as a stepwise process, with one task leading to the next one sequentially. However, optimizing a DAP and data workflows is generally an iterative process of learning from trial and error. Perhaps the first round of data collected in the community will not be sufficient to answer the questions of the community action plan, or the data dissemination plan will not result in end-users accessing or being able to use the data they need. Community partners might innovate workflow ideas not outlined in the plan, such as by finding low-tech solutions where high-tech ones did not work or creating alternate visualizations of data that more appropriately represent the data being monitored. These innovations should spark a revisit of prior steps, including changing the DAP to ensure that all activities remain well-synchronized with community goals and workflows as well as data governance decisions.

## Methods

HCS researchers in Ohio developed and implemented the CEDS model as a tool to engage community coalitions in DDDM as part of HCS. HCS was a multisite, wait-listed, community-level cluster-randomized trial that sought to test a community-level intervention, called Communities That HEAL (CTH), in 67 communities across four state-level sites (Kentucky, Massachusetts, New York, and Ohio) [36–40]. The CTH involved engagement with community coalitions to promote a common vision, shared goals, and selection of tailored EBP strategies using data-driven community response plans across multiple sectors to reduce opioid overdose deaths and associated outcomes.

Data was instrumental in the HCS, from helping communities make informed decisions between a selection of EBPs that served their needs to monitoring implemented practices for community and research purposes. HCS defined some cross-site parameters for study-level data collection and public use, discussed elsewhere [41, 42], however each site had latitude to determine how to engage communities in data activities. In Ohio, the research team developed and implemented CEDS using data ambassadors to work closely with the communities in data aspects of the CTH.

The “HCS Ohio” site selected 19 non-contiguous counties affected by the opioid epidemic and randomized them to the first wave and the wait-listed controlled second wave. One county declined to participate prior to implementation of the intervention. The nine Wave 1 counties received the CTH intervention from January 2020 through June 2022; the nine Wave 2 counties received the CTH from July 2022 to December 2023.

HCS Ohio employed a three-person field team to work with each county coalition: a community engagement facilitator who coordinated overall community engagement; an implementation facilitator who had expertise with the EBPs; and a community data coordinator. The community data coordinator’s (or data ambassador in CEDS) responsibility was to coordinate with the field team, the academic data scientists, coalition members, and community partners to facilitate the use of data to support EBP selection and outcomes monitoring. Data ambassadors used the CEDS steps to guide their activities within the HCS.

In early 2024 after the CTH intervention concluded, seven data ambassadors, including two who became managers of multiple community data ambassadors during the study period, and a data manager working with communities during Wave 1 and Wave 2 of HCS reflected on their experiences using CEDS in the HCS. Each ambassador provided written and/or oral responses on each CEDS step, sharing examples of success and failure. We compiled these examples and have highlighted shared lessons learned that were found across ambassadors’ reflections.

## Results: Using CEDS to Address the Opioid Epidemic and Lessons Learned

### Lesson 1: Relationship Building is Key

Data ambassadors were hired because of their interpersonal and data skills as well as knowledge of communities. Many of the data ambassadors lived and worked in the community in which HCS hired them to work, had prior knowledge of the coalition partners and data landscape, and often had experience working on the topic of addiction and mental healthcare. Coalitions gave input on hiring decisions of the data ambassador and other field team members. In some communities, the interactions between the community and the data ambassador were hampered, often due to factors such as timing of the study, study priorities, and deadlines. In communities where a data champion and/or a data workgroup built a strong relationship with the data ambassador, the ability to design a DAP and put data into action was faster, required fewer resources, and had a greater impact. For example, one data ambassador was hired after working as a data analyst for the public health department, a key coalition partner which facilitated designing the exemplar DAP. However, in other communities, as one data ambassador noted, “there was gatekeeping about relationship development” that hampered her ability to fully implement CEDS.

### Lesson 2: Importance of Timing of Data Inclusion in Decisions

Data use in CEDS is framed as a process to achieve goals guided by a community vision, so data use is delayed until a shared vision and set of goals begin to form. However, broaching data as part of decision making for community change needs to occur early and often. The CTH intervention involved phased work and, during Wave 1, data activities were scheduled to be implemented at a later phase in the intervention. Because of this scheduling, data ambassadors were limited in when and for what purpose they could have data discussions. One data ambassador mentioned that having limited access to community action planning details hindered her ability to incorporate the community vision and goals into her data action planning work. Notably, Wave 2 incorporated lessons from the challenges of implementing data engagement models like CEDS when adhering to the original Wave 1 phased schedule. Data work was included earlier in the Wave 2 CTH process which facilitated moving through the CEDS steps as designed. For example, in a rural Wave 2 community, a coalition goal (CEDS Step 1) to prescribe medication for opioid use disorder (MOUD) that faced some strong community resistance, was eventually implemented based on compelling data (CEDS Step 6) that was shared early enough to influence community members’ orientation to the need for such a goal.

### Lesson 3: Communities do not Need Sophisticated Data Use Experience to Make Profound Use of Data

HCS Ohio communities often lacked resources and/or experience using integrated data systems, yet this did not mean those communities were fundamentally incapable or disinterested in using data. Most communities, in whole or in part, had interest in learning what to do with preexisting data for DDDM. Data ambassadors found that both rural and urban communities often suffered from disjointed data collection and lack of cross-sector communication and coordination of the data they had. In one urban community, the data ambassador was told by a key coalition member that the people in the community do not trust computers, so data should be collected on paper. Notably, this community also had access to sophisticated data systems and experienced data analysts. Contrastingly, in multiple rural communities, the coalition wanted to learn about the opioid crisis through data but did not have resources to begin the process without the kind of concentrated effort and resources HCS Ohio was able to provide. The addition of a data ambassador in one community enabled connections between a hospital, EMS, and other first responders to leverage existing data in a novel manner across sectors, resulting in a community-facing data dashboard that tracked behavioral health and substance use disorder population outcomes that the community desired.

### Lesson 4: No Individual Data Science Tool Universally Served Every Community’s Data Needs

As part of the CTH, each site developed a community-facing data dashboard with the intent that the data could be used for DDDM and implementation monitoring in HCS selected communities [42]. HCS Ohio data ambassadors were trained on and presented data from these dashboards to their respective communities. These dashboards showed data at a county level and often were presented with a 6-months lag. The dashboards were not available at the beginning of Wave 1, which contributed to further challenges for communities to use these data dashboards in a timely manner. During the CEDS process, data ambassadors found that the HCS-provided data was rich yet was not granular enough to serve the communities DAP needs. Instead, most of the DAPs involved incorporation of local data that could represent what was happening at a township or neighborhood level within the past month, week, or day. For example, in one community where they were excited about the data from HCS, they also needed more detailed, community-tailored data. The data ambassador explained that there was a lot of disparate data on community naloxone (a medication to reverse opioid overdoses) distribution leading to over-saturation in low-risk areas and under-distribution in areas with high rates of opioid overdoses. The data ambassador worked collaboratively to create a sustainable process to collect the data from disparate data sources in one location, share the combined data to the coalition, which then was used to target future naloxone distribution to areas of greatest need.

### Lesson 5: CEDS is Most Valuable When Used Flexibly and Iterated

The data ambassadors understood that the CEDS process was flexible and should allow for revision of earlier steps and processes. However, this became even clearer in practice as data ambassadors needed to be receptive to changing understandings of communities’ needs. One data ambassador had experience in a Wave 1 community building a REDCap [43] database to track community naloxone distribution as part of the CEDS process. In Wave 2, she worked with a different community that also decided to track naloxone, and their original DAP included a REDCap database. The data ambassador built and tried to implement a REDCap system, but this system did not work for the community. After revision of the CEDS steps, she developed a Smartsheet system, which also failed to meet the community’s needs. Eventually, the community decided on use of Microsoft Forms for data collection which they knew would be usable and sustainable. In hindsight, the data ambassador learned that the REDCap system in her Wave 1 community was not sustained because of staffing issues, yet the system developed through trial-and-error in Wave 2 was sustained.

### Lesson 6: Every Application of CEDS was Imperfect, Yet Worthwhile

Data ambassadors discussed the usefulness of the CEDS model to bring data to use in communities, even as they experienced many barriers to implementing the ideal model. Data ambassadors in Wave 1 were constrained by COVID-19 and communities’ divided attention. In some communities, other funded projects limited data work and divided coalition efforts. Funding, staffing changes, and data access often delayed or derailed DAP before data ambassadors and coalitions could move data into action. However, each data ambassador was able to describe examples in which at least some aspect of the CEDS model worked to change an organization or a coalition’s use of data for decision making, leading to more thoughtful processes or outcomes consistent with community vision and goals.

## Conclusion

Many communities understand the value of using data to make decisions to drive community change. However, while tremendous volumes of data now exist that touch on issues of interest to communities, this has created a tendency at the local level to transform data-driven decisions to fit the topics data can address rather than transforming the data system to address community needs. The CEDS model seeks to encourage communities to put their vision and goals at the center of a data science process and design data systems that support community goals. In many cases, communities will find through community data landscape assessment that there are locally available data and local entities with realized or unrealized data potential that can be leveraged to support community goals.

CEDS was developed to support the selection, monitoring, and evaluation of evidence-based practices through a community-engaged process to tackle the effects of substance use and addiction on communities. Although the model was developed within a public health framework, CEDS was designed to be applicable to diverse challenges communities face across many sectors (e.g., transportation, housing, homelessness, gentrification). CEDS fills a gap by providing guidance on how to collaborate to use data to select appropriate intervention strategies, monitor implementation of those strategies, and assess outcomes as they relate to community goals.

## Supplementary Information

Below is the link to the electronic supplementary material.

Supplementary file1 (DOCX 24 KB)

## Data Availability

The author confirms that all data generated or analyzed during this study are included in the manuscript.
