# Supplementary material for: Community-Engaged Data Science (CEDS): A Case Study of Working with Communities to Use Data to Inform Change
Source: J Community Health. 2024 Jul 3;49(6):1062–72. doi: 10.1007/s10900-024-01377-y (PMC11413031; doi:10.1007/s10900-024-01377-y)
Supplement: Supplementary file 1 — Supplementary file1 (DOCX 24 KB) [file 10900_2024_1377_MOESM1_ESM.docx]

**Appendix 1**

**Community Data Landscape and Data Visualization Interview Tool**

**PURPOSE OF ACTIVITY:** *Understand who is engaged in current data activities in the community; Create a data wish list; Identify participants for data landscape interviews. Please use the following data topics relevant to your situation*.

**CURRENT DATA REPORTING AND DATA CURATION:**

- What data (are you currently collecting)/(have you collected in the past)?
  - [For health institutions] Do you collect data for Community Health Assessment (CHA)/Community Health Improvement Plan?
  - What is the process for acquiring and analyzing these data?
    - *PROBE for differences processes by data source.*
  - Who is involved in this process? Who is the lead for the data-related portion of the plan?
- What other reporting or surveillance activities does your community engage in that require data?
  - What is the purpose of these activities? How often do they happen?
  - What is the process by which data are requested and acquired? What organizations are involved? Who are the key players?
  - Who leads the data analysis? Reporting activities?
  - Who has access to the data used for reporting? Who has access to the final reports?
- What barriers have you encountered in conducting regular reporting (e.g., financial, capacity, staff, etc.)?

**FOR SUBSTANCE USE AND MENTAL HEALTH SPECIFIC DATA:**

- What data related to opioid use or substance use and mental health have you used? Where did these data come from?
- Is there data/information on this list that you are especially interested in? Are there other data not on this list you would like to have to help you make a decision about the EBPs?
  - What additional data would you like to have on at risk populations? Settings? MOUD? Safe prescribing practices?

**ADDITIONAL DATA RESOURCES:**

- If you could construct a “data wish list”, what would be on it?
- What data do you not have access to that would be nice to have for making decisions?
- Are there any initiatives in your community that would benefit from sharing (data) resources?
  - What barriers are encountered for these activities?
  - How might organizations benefit from sharing or combining data?
  - Are there shared values and goals around use of data across organizations?
  - *PROBE: Ask about goals such as financial, regulatory, human capital, daily operations, surveillance.*

**ORGANIZATION-SPECIFIC DATA:**

- How does your organization use data to:
  - provide services?
  - set goals?
  - plan programs?
  - evaluate impact/effectiveness?
- What are the biggest challenges your organization faces in acquiring and using data?
- Who at your organization is "in charge" of data? Who provides access to data, analyzes data, or produces reports?
- Is there anything your organization would like to accomplish that it currently cannot that data might help with?
- Do you currently have any data sharing activities with other organizations, businesses, or public health entities in the community?
  - What are/have been the barriers/enablers sharing data?

**Snowball sampling:**

- Are there specific sectors or organizations that have not been involved in reporting, surveillance or data sharing activities traditionally, that you think should?
- Who are the 3 people I need to talk to about the data resources in this community?
